# Exploring the efficacy of laser speckle contrast imaging in the stratified diagnosis of rosacea: a quantitative analysis of facial blood flow dynamics across varied regions

**DOI:** 10.3389/fimmu.2024.1419005

**Published:** 2024-08-23

**Authors:** Jin-Feng Liao, Xue-Mei Jiang, Zhen Xie, Hua Lei, Juan Luo, Yu Lv, Gang Liu, Yujie Mao, Si-Yuan Song, Yi Wang

**Affiliations:** ^1^ Department of Dermatology, Sichuan Provincial Peoples Hospital, University of Electronic. Science and Technology of China, Chengdu, China; ^2^ Healthcare-associated Infection Control Center, Sichuan Academy of Medical Sciences, Sichuan Provincial People’s Hospital, School of Medicine, University of Electronic Science and Technology of China, Chengdu, China; ^3^ Department of Neuroscience, Baylor College of Medicine, Houston, TX, United States; ^4^ Clinical Immunology Translational Medicine Key Laboratory of Sichuan Province, Center of Organ Transplantation, Sichuan Academy of Medical Science and Sichuan Provincial People’s Hospital, Chengdu, China

**Keywords:** rosacea, Think View, LSCI, facial blood perfusion, dermatological assessment, ETR, PPR, multispectral imaging analyzer

## Abstract

**Background:**

Rosacea has a high incidence, significantly impacts quality of life, and lacks sufficient diagnostic techniques. This study aimed to investigate the feasibility of laser speckle contrast imaging (LSCI) for measuring facial blood perfusion in patients with rosacea and to identify differences in blood flow among various facial regions associated with different rosacea subtypes.

**Methods:**

From June to December 2023, 45 patients were recruited, with 9 excluded, leaving 36 subjects: 12 with erythematotelangiectatic rosacea (ETR), 12 with papulopustular rosacea (PPR), and 12 healthy controls. The Think View multispectral imaging analyzer assessed inflammation via gray reading values across the full face and five facial areas: forehead, nose, cheeks, and chin. LSCI measured and analyzed blood perfusion in the same areas. Plasma biomarkers interleukin-6 (IL-6), IL-1β, and tumor necrosis factor-α (TNF-α) were tested in different groups.

**Results:**

Both ETR and PPR groups showed increased average blood perfusion and facial inflammation intensity by gray values compared to controls, with statistically significant differences. Average blood perfusion of ETR and PPR groups showed increased values in the forehead, cheeks, and nose, compared to controls, and the values in the cheeks were statistically different between ETR and PPR. The facial inflammation intensity of the ETR group showed increased values in the forehead and cheeks, and the PPR group showed increased gray values in the forehead, cheeks, nose, and chin compared to controls, and the values for the cheeks, nose, and chin were statistically significantly different between ETR and PPR. Plasma biomarkers IL-6, IL-1β, and TNF-α were significantly elevated in both ETR and PPR groups compared to controls.

**Conclusion:**

LSCI is a valuable, non-invasive tool for assessing blood flow dynamics in rosacea, providing a data foundation for clinical research. Different rosacea subtypes exhibit distinct lesion distribution and blood flow patterns, and both ETR and PPR could affect all facial areas, particularly the cheeks in ETR and the forehead, nose, and chin in PPR.

## Introduction

Rosacea is a complex skin condition mainly affecting the face, characterized by redness, swelling, and vascular changes that impair life quality (1–5). The disorder’s exact cause is unclear, involving immune, neurovascular, and glandular systems (2, 3, 6, 7). Various triggers, such as sunlight, hot weather, alcohol, spicy foods, environmental factors, *Demodex* mites, and stress, contribute to symptom exacerbation (8–10).

The original standard classification of the rosacea report of the National Rosacea Society Expert Committee 2002 identified the most common patterns or groupings of signs and symptoms and designated them as follows: subtype 1, erythematotelangiectatic; subtype 2, papulopustular; subtype 3, phymatous; and subtype 4, ocular (11). Because rosacea can encompass a multitude of possible combinations of signs and symptoms, the following updated classification system (2017) is based on phenotypes to provide the necessary means of assessing and treating rosacea in a manner that is consistent with each individual patient’s experience (12): For the diagnosis of rosacea, at least one diagnostic or two major phenotypes are required. The diagnostic criteria include fixed centrofacial erythema in a characteristic pattern that may periodically intensify and phymatous changes. The major phenotypes are flushing, papules and pustules, telangiectasia, and ocular manifestations such as lid margin telangiectasia, interpalpebral conjunctival injection, spade-shaped infiltrates in the cornea, scleritis, and sclerokeratitis.

Current diagnostic approaches, including dermoscopy and capillaroscopy, offer limited quantitative analysis (13–16), while other methods like laser Doppler velocimeter (LDV) are constrained by their operational complexity (17, 18). LDV is one of the earliest technologies used to detect skin blood flow in patients with rosacea. Studies have found that patients with rosacea have more abundant skin blood flow compared to control groups (19). LDV is based on the Doppler frequency shift, which is the change in frequency of the laser light reflected by moving red blood cells, simulating real-time perfusion blood flow (20). This technique requires the use of a fiber-optic probe and laser to contact the skin, and it cannot simultaneously present the overall facial image, necessitating separate measurements of vascular perfusion in different areas, which can significantly interfere with the results.

Thus, there is a need for precise, non-invasive diagnostic tools to measure skin blood flow accurately.

## Materials and methods

### Patient samples

Patient information: A total of 45 people were initially recruited; however, 3 people were excluded because of medication history within 1 week, 3 people were excluded because they refused to have their blood tested, and 3 people were excluded because they experienced acute dermatitis flare-ups. This left 36 participants for the study, with 12 in each group. All 36 participants provided informed consent. A total of 24 patients with rosacea from Sichuan Provincial People’s Hospital were from outpatient visits from 1 June to 31 December 2023. Among these, 12 cases were classified under the ETR group, all of whom were women. In the PPR cohort, there were 10 female and 2 male patients. A distribution of gender difference in PPR group showing significance by chi-square test (*p* < 0.05). Similarly, the control group consisted of 10 female and 2 male patients, also showing significance by chi-square (*p* < 0.05). None of the female patients were in their menstrual period at the time of the study, and the time since their last menstrual period was recorded. No medication was used within 1 week before the examination. However, medication was intermittently used previously: ETR: All patients used repair-type skincare products topically. Combined use (hydroxychloroquine 200 mg po bid + carvedilol 6.25 mg po bid, *n* = 4) and single use (hydroxychloroquine 200 mg po bid, *n* = 4). PPR: Combined use (doxycycline 40 mg po qd + metronidazole gel, topical bid, *n* = 8) and single use (metronidazole gel, topical bid, *n* = 4) (**Table 1**).

**Table 1:** Demographic and clinical findings of patients with rosacea and healthy control subjects.

Variable	PPR	ETR	NORMAL	P
Age, year, mean ±SD	34.67±10.86	32.33±4.91	36.92±10.27	0.4738
Men/women,n	3/9	0/12	1/12	
Days since last menstrual period, d	9.33±2.92	9.08±3.0	9.19±3.07	
Fitzpatrick skin type, n				
II	2	8	0	
III	8	4	7	
IV	2	0	5	
Age of onset of rosacea, mean ± SD	27.96±6.28	29.63±4.74	-	0.4708
Disease duration,mean ± SD	6.71±6.12	2.67±0.83	-	0.0335*
Disease severity,n				
Mild	4/12	4/12	-	
Moderate	4/12	4/12	-	
Severe	4/12	4/12	-	
Eye involvement, n				
Yes	0	0		
No	12	12		
Family history of cardiovascular events, n	0	0		
BMI, Kg/m2, mean ±SD	22.27±2.00	20.85±1.16	21.80±0.94	0.0793
Systolic BP	112.17±6.07	107.92±4.17	107.33±4.36	0.4228
Diastolic BP	68.42±4.96	67.25±3.52	67.08±3.40	0.6793
Autoimmune Diseases				
SLE	0	0		
RA	0	0		
Dermatomyositis	0	0		
Urticarial vasculitis	0	0		
Ankylosing spondylitis	0	0		
Malignant tumors	0	0		
Infectious diseases	0	0		

* P<0.05

Ethical approval for this study was granted by the Medical Ethics Committee of Sichuan Provincial People’s Hospital, under the approval number 2023-276. In accordance with the Declaration of Helsinki, informed consent was obtained from all patients, allowing the use of their images and serological data for this research.

Inclusion criteria: These are based on the original standard classification of rosacea report of the National Rosacea Society Expert Committee 2002, classified into the ETR group and the PPR group (11). Participants were aged 18–70 years and did not have any other major systemic diseases. The healthy control group consisted of individuals seeking cosmetic consultations at our hospital. Clinical severity of skin lesions was graded by physicians using the NRSS grading system (21).

Exclusion criteria included the following: (1) hormone-dependent dermatitis, seborrheic dermatitis, or other skin inflammations; (2) facial burns or traumatic scars; (3) psychiatric disorders or severe cognitive impairments; (4) lactating or pregnant women; (5) coexisting malignant tissue or organ diseases; (6) use of oral and topical medications within the past week; (7) participation in other studies simultaneously; and (8) smokers and coffee drinkers.

### Detection instruments

Our study utilized advanced multispectral imaging systems for the detailed analysis of cutaneous conditions and employed a high-resolution multispectral imaging analyzer that captures a wide array of skin features, significantly enhancing diagnostic processes.

Additionally, we incorporated a sophisticated LSCI apparatus to non-invasively measure and map facial blood flow and perfusion, which is vital in our investigation of microcirculatory dynamics.

### Think View multispectral imaging analyzer

Think View (manufactured by Wuhan Bose Electronic Co. Ltd., production number: 2120000038) features an imaging resolution of 2,000 × 3,000, with a horizontal resolution of 96 dpi and a vertical resolution of 96 dpi. This is a color imaging system that employs multispectral hybrid scanning technology, including parallel polarized light imaging, cross-polarized light imaging, hybrid near-infrared (NIR) light imaging, NIR light imaging, ultraviolet light imaging, and brown light imaging.

Participants’ basic information was input; they were asked to wear a black shawl and black headband, close their eyes, and relax throughout the process. The patient’s head was positioned at the designated spot on the headrest for frontal facial imaging. The focus was locked, ensuring the facial edges and nose overlapped with the green frame in the image; real-time images were saved for later comparison and analysis.

The system captures images of patients’ faces and automatically diagnoses lesion areas, without the capability to detect inflammation concentration in specific areas, such as telangiectasia and papules/pustules, separately. Therefore, we converted the NIR detection images to grayscale and used ImageJ software to select the larger inflammation areas to more comprehensively cover the five facial areas—the forehead, nose, bilateral cheeks, and chin—for quantification. After selecting the areas, they can be directly applied to the same areas of another patient, thus obtaining the related gray values. The lower the grayscale value, the stronger the inflammatory intensity.

### Laser speckle contrast imaging device

LSCI (manufactured by Perimed AB, Sweden, Model PeriCam PSI NR) uses an invisible NIR laser (785 nm) for blood perfusion measurements. LSCI could follow the kinetics of the blood response at a rate of up to 90 frames per second, with an imaging resolution of 2,448 × 2,048, and the results are displayed in perfusion units. During the monitoring of blood perfusion, when moving objects, such as red blood cells in tissues, are illuminated by the laser, the backscattered light forms a speckle pattern composed of dark and bright areas, thereby indicating different levels of blood perfusion (22). Additionally, after speckle imaging, any area of the face can be selected for blood perfusion analysis to more accurately and intuitively observe the microvascular blood flow distribution.

The basic information of the participants was recorded. The participant’s face was positioned directly in front of the laser speckle blood flow imaging device’s probe (camera). The software operation page was opened, and the observation area was selected and focused. After selecting the observation area, the laser was turned on, indicated by the laser light. Custom exposure times were set, and the device was switched to flow speed image mode for simultaneous observation of original speckle images and flow speed images that were saved. The larger inflammation areas were selected to more comprehensively cover the five facial areas: the forehead, nose, bilateral cheeks, and chin for quantification. After selecting the areas, they can be directly applied to the same areas of another patient to record blood flow distribution. The analysis results were presented in “pseudocolor” images, where colors were assigned based on flow speed values—red for relatively high flow speed, blue for low flow speed, and green for medium flow speed—to analyze changes in blood flow in the respective parts.

On day 7 after drug abrogation, we simultaneously conducted tests for interleukin-1β (IL-1β), IL-6 (**Table 2**), and tumor necrosis factor-α (TNF-α) after the equipment detection was completed. For the serum pro-inflammatory cytokine detection, commercial enzyme-linked immunosorbent assay (ELISA) kits were used according to the manufacturer’s instructions. Briefly, patient serum was collected and incubated with specific antibodies coated on a microplate. After washing away unbound substances, an enzyme-linked secondary antibody specific for the cytokine was added to each well. Following a second washing step to remove unbound antibody-enzyme reagent, a substrate solution was added to the wells to produce a color change reflective of the cytokine levels. The color development was halted with the addition of a stop solution, and the intensity of the color was measured at a specific wavelength using a microplate reader. The cytokines were quantified using the following ELISA kits: Human IL-6 ELISA Kit (Catalog No. ABC123), Human IL-1β ELISA Kit (Catalog No. DEF456), and Human TNF-α ELISA Kit (Catalog No. GHI789). Each sample was assessed in duplicate to ensure accuracy, and cytokine concentrations were calculated based on a standard curve prepared using known concentrations of human cytokines. Data were expressed in picograms per milliliter (pg/mL).

**Table 2:** Patient discontinued prior to concomitant Other medication use.

Variable	Hydroxychloroquine 200mg po bid	carvedilol 6.25mg po bid	Restorative Skin Care bid	doxycycline 40mg po qd	Metronidazole Gel bid
Patient E1	√	√	√	-	-
Patient E2	√	√	√	-	-
Patient E3	√	√	√	-	-
Patient E4	√	√	√	-	-
Patient E5	√	-	√	-	-
Patient E6	√	-	√	-	-
Patient E7	√	-	√	-	-
Patient E8	√	-	√	-	-
Patient E9	-	-	√	-	-
Patient E10	-	-	√	-	-
Patient E11	-	-	√	-	-
Patient E12	-	-	√	-	-
Patient P1	-	-	-	√	√
Patient P2	-	-	-	√	√
Patient P3	-	-	-	√	√
Patient P4	-	-	-	√	√
Patient P5	-	-	-	√	√
Patient P6	-	-	-	√	√
Patient P7	-	-	-	√	√
Patient P8	-	-	-	√	√
Patient P9	-	-	-	-	√
Patient P10	-	-	-	-	√
Patient P11	-	-	-	-	√
Patient P12	-	-	-	-	√

### Detection environment

All tests were conducted in a controlled room temperature (25 ± 2) °C, with relative humidity (RH) (55 ± 3)%. The facial skin was cleaned with a facial cleanser and kept dry; participants were asked to avoid vigorous exercise, emotional fluctuations, smoking, drinking coffee, and other factors that might aggravate erythema 30 min before testing and to rest quietly for 10 min before imaging. All patients were first observed using the Think View, followed by the LSCI.

### Statistical analysis

Data were analyzed using the statistical software GraphPad Prism version 9.5.0. Quantitative data were presented as mean ± standard deviation. To compare the two groups, we utilized an unpaired *t*-test. *p* < 0.05 was considered statistically significant.

## Results

In our comprehensive analysis, we have carefully delineated the nuanced variations in facial inflammation intensity and blood perfusion across distinct facial regions in patients with ETR and PPR.

**Figure 1:**
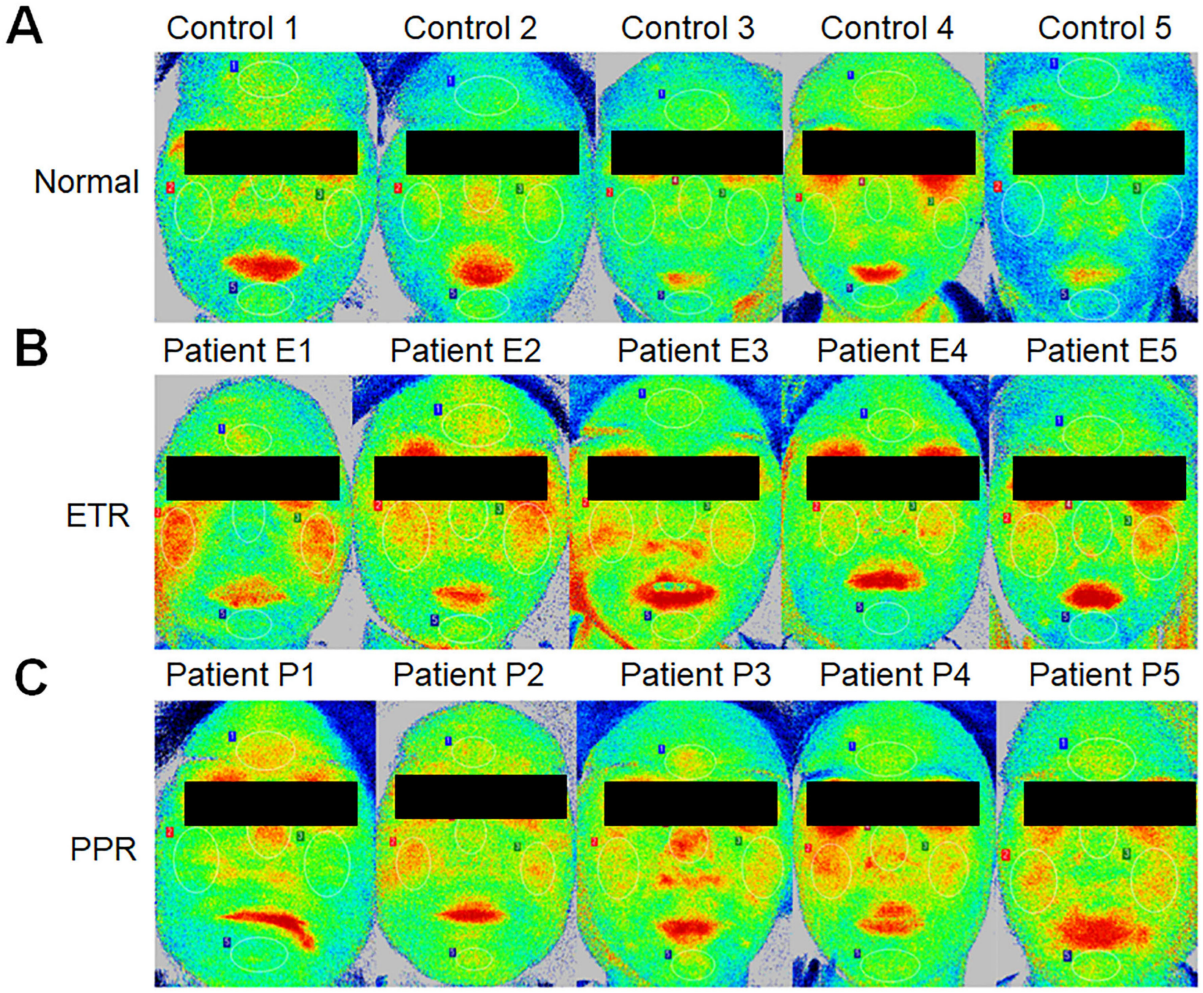
Laser speckle contrast imaging (LSCI) analysis of facial blood flow. **(A)** Control group showing typical baseline perfusion. **(B)** Patients with erythematotelangiectatic rosacea (ETR) demonstrating increased facial blood flow, particularly in regions commonly affected by the subtype. **(C)** Patients with papulopustular rosacea (PPR) displaying patterns of perfusion correlating with the inflammatory activity characteristic of this rosacea subtype.

**Figure 2:**
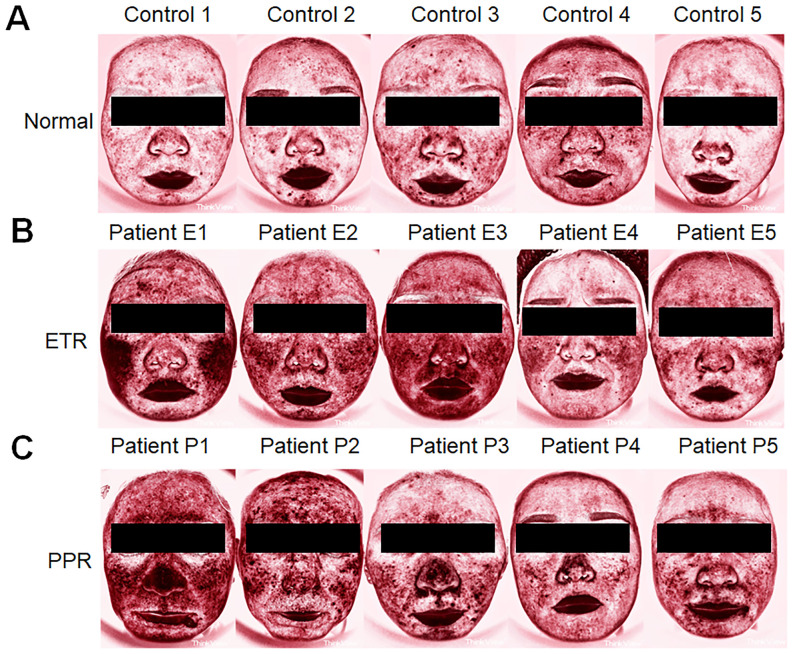
Comparative Think View facial analysis in rosacea. **(A)** Faces of control individuals without rosacea features, serving as a baseline for comparison. **(B)** Faces of patients with ETR, showing the typical erythema and vascular features associated with the subtype. **(C)** Faces of patients with PPR, marked by the presence of papules and pustules in addition to erythematous changes.

**Figure 3:**
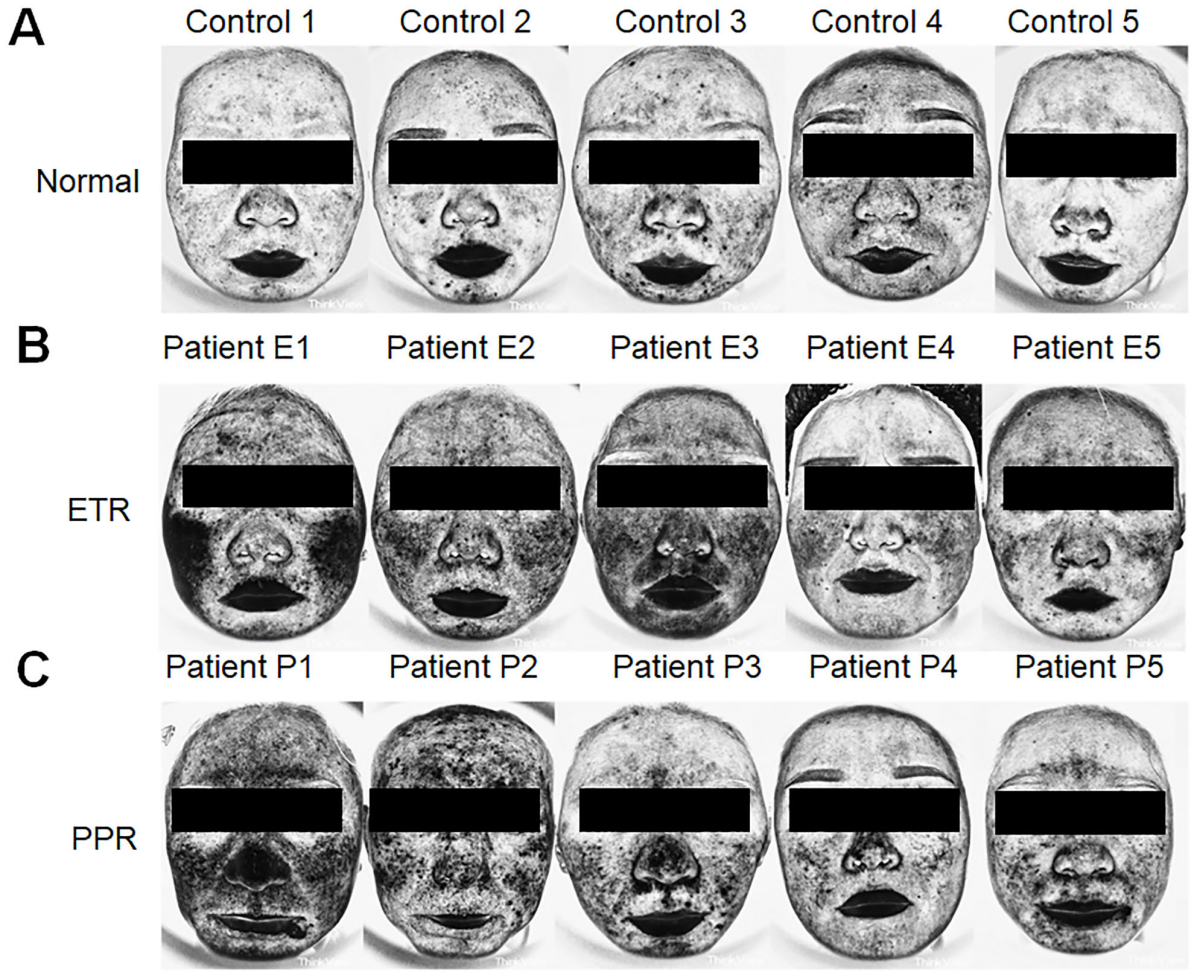
Assessment of inflammation intensity using Think View imaging. **(A)** Normal controls with minimal inflammatory indications. **(B)** Patients with ETR exhibiting inflammation patterns consistent with erythematotelangiectatic presentations. **(C)** Patients with PPR revealing inflammation associated with papular and pustular lesions, along with erythema.

Additionally, when assessing skin condition through Think View and LSCI, a correlation between clinical observations and imaging data was established, as shown in the LSCI image (**Figure 1**), Think View NIR detection image (**Figure 2**), and the NIR detection image displayed in grayscale (**Figure 3**).

### Differential analysis of facial blood perfusion in rosacea subtypes by LSCI

**Figure 4:**
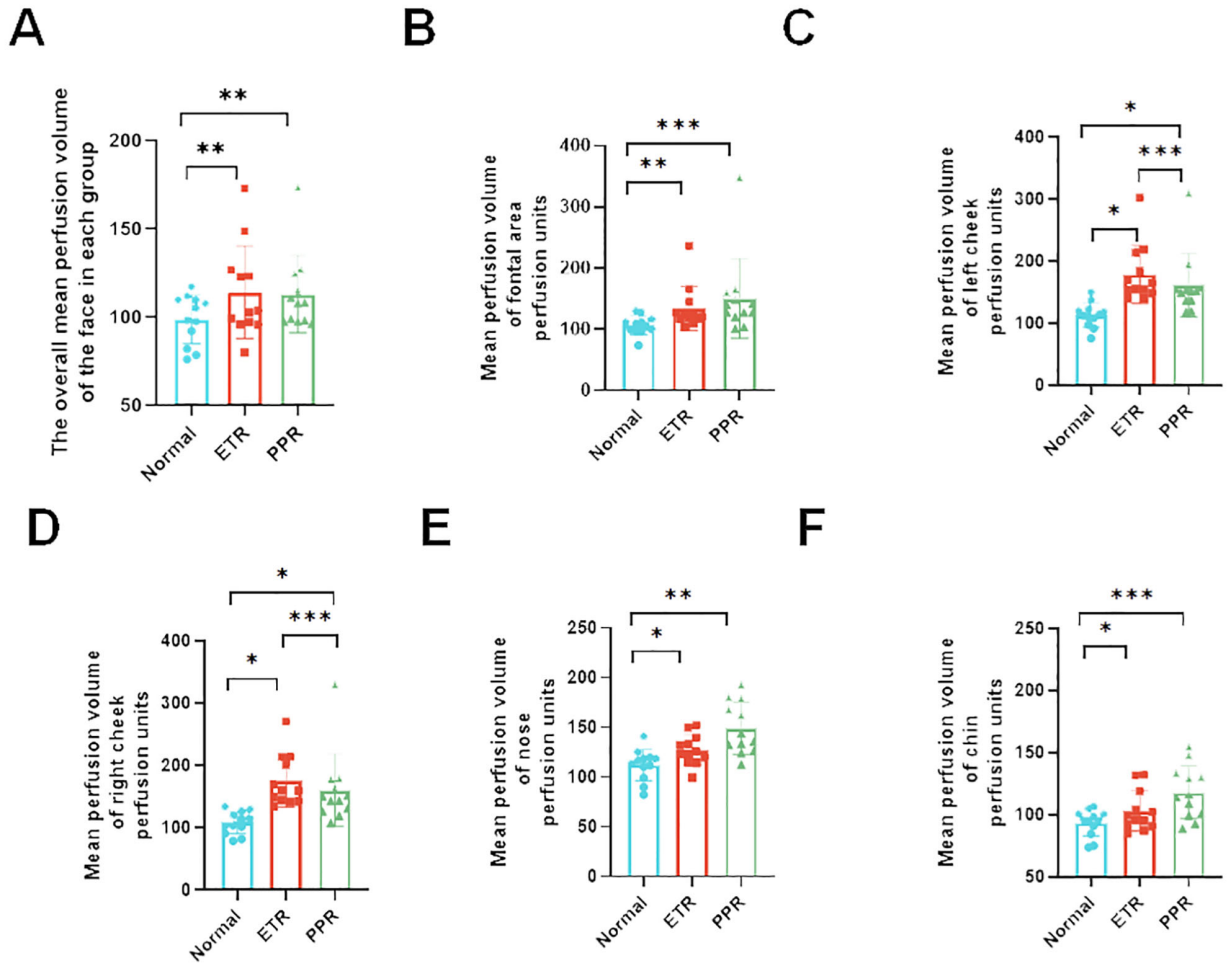
Analysis of blood perfusion volumes in various facial regions among the control, ETR, and PPR groups. The blood perfusion volume is consistent across all groups with some variations. **(A)** Overall facial perfusion (vs. control, ETR, and PPR *p* < 0.01), **(B)** forehead (vs. control and ETR *p* < 0.01, PPR *p* < 0.001), **(C)** left cheek (vs. control, ETR, and PPR *p* < 0.05, between ETR and PPR, *p* < 0.001), **(D)** right cheek (vs. control, ETR, and PPR *p* < 0.05, between ETR and PPR, *p* < 0.001), **(E)** nose (vs. control and ETR < 0.05, PPR *p* < 0.01, not significant between ETR and PPR), and **(F)** chin (vs. control and ETR < 0.05, PPR *p* < 0.001, not significant between ETR and PPR). *P<0.05, **P<0.01, ***P<0.001.

**Figure 5:**
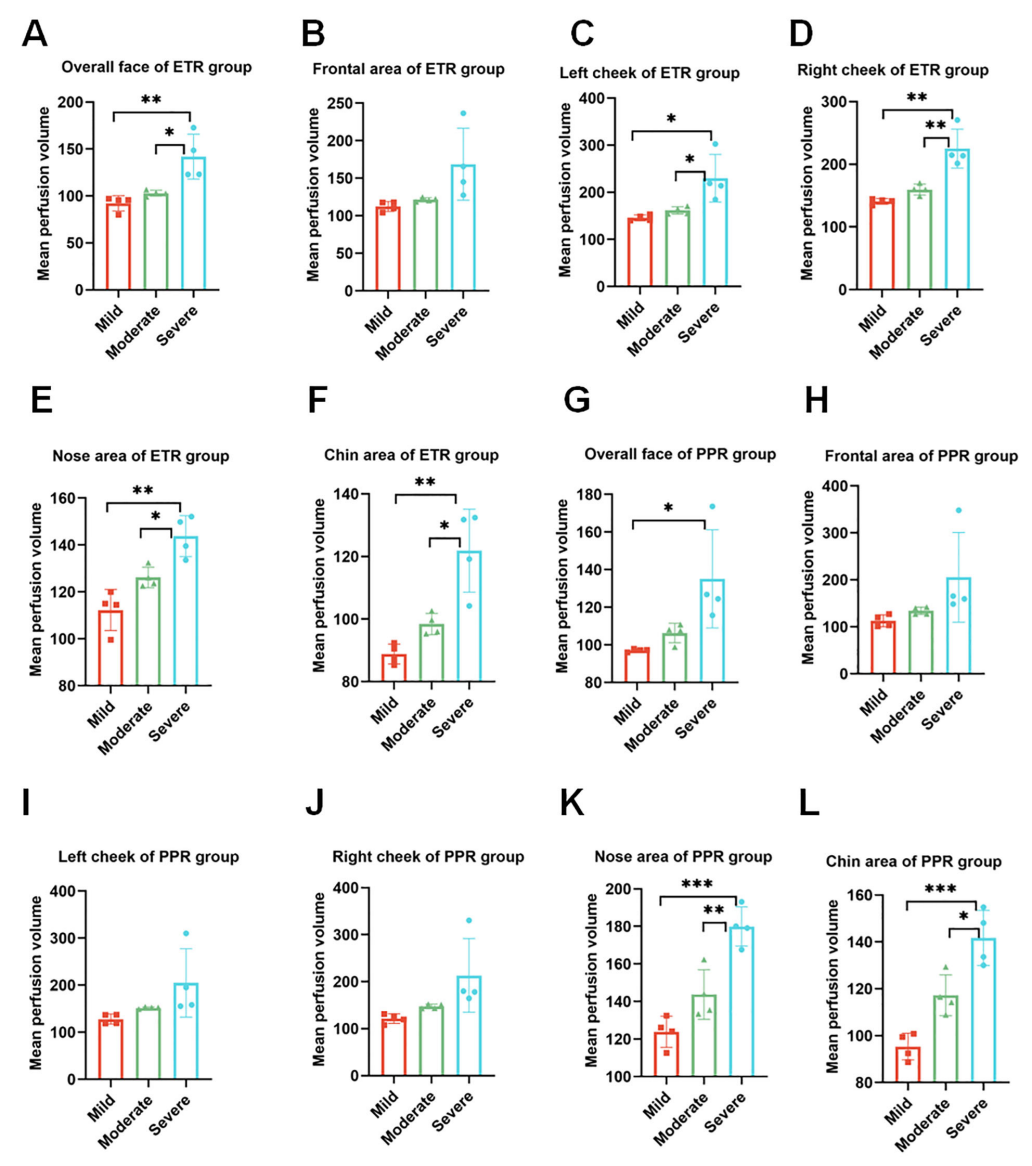
Analysis of blood perfusion volumes in the mild, moderate, and severe regions. **(A–F)** Overall facial perfusion, left cheek, right cheek, nose, and chin areas of the ETR group among mild, moderate, and severe participants. **(G–L)** Overall facial perfusion, left cheek, right cheek, nose, and chin areas of the PPR group among mild, moderate, and severe participants. *p < 0.05; **p < 0.01; ***p < 0.001.

In our comprehensive analysis of full facial blood flow, we observed significant differences among the groups studied. Specifically, the normal group exhibited an average blood flow of 86.27 ± 13.34, while the ETR group showed a marked increase to 112.28 ± 25.98, and the PPR group demonstrated a similar elevation at 112.85 ± 21.91. These differences in ETR and PPR are statistically significant (*p* < 0.01) when compared to the normal group, and there is no statistically significant difference between the ETR and PPR groups. Specifically, the blood perfusion on the forehead of the ETR group showed a significant increase (*p* < 0.01), and the PPR group exhibited a similar increase (*p* < 0.001). There is no statistically significant difference between the ETR group and the PPR group. In the case of the left and right cheek, a significant increase was observed in the ETR group compared to the normal group (*p* < 0.05), with the PPR group also showing an increase (*p* < 0.05). Notably, the increase in the ETR group was more pronounced than in the PPR group (*p* < 0.001). On the nose, the ETR displayed significant increases compared to the normal group (*p* < 0.05), and PPR groups exhibited a similar increase (*p* < 0.01). There is no statistically significant difference between the ETR group and the PPR group. The chin area of the ETR group indicated an increase when compared to the normal group (*p* < 0.05). The PPR group, on the other hand, showed a markedly significant increase (*p* < 0.001) (**Figure 4**). Lastly, we compared the various mean perfusion areas of mild, moderate, and severe patients, including the overall facial, frontal, left cheek, right cheek, nose, and chin areas of patients with ETR (**Figures 5A–F**) and patients with PPR (**Figures 5G–L**).

### Differential analysis of facial inflammation intensity in rosacea subtypes by gray values

Our study found that the darker the NIR image of Think View is, the lower the gray value is. It represents the stronger facial inflammation intensity. The normal group exhibited gray values of 183.77 ± 13.33, while the ETR group showed a marked decrease to 171.15 ± 12.81, and the PPR group demonstrated a similar decrease to 165.51 ± 20.10. These differences in ETR and PPR are statistically significant (*p* < 0.01) when compared to the normal group. There is no statistically significant difference between the ETR and PPR groups. Specifically, the gray values on the forehead were diminished, with the ETR group showing a significant decrease (*p* < 0.05) and the PPR group exhibiting a similar reduction (*p* < 0.05).

**Figure 6:**
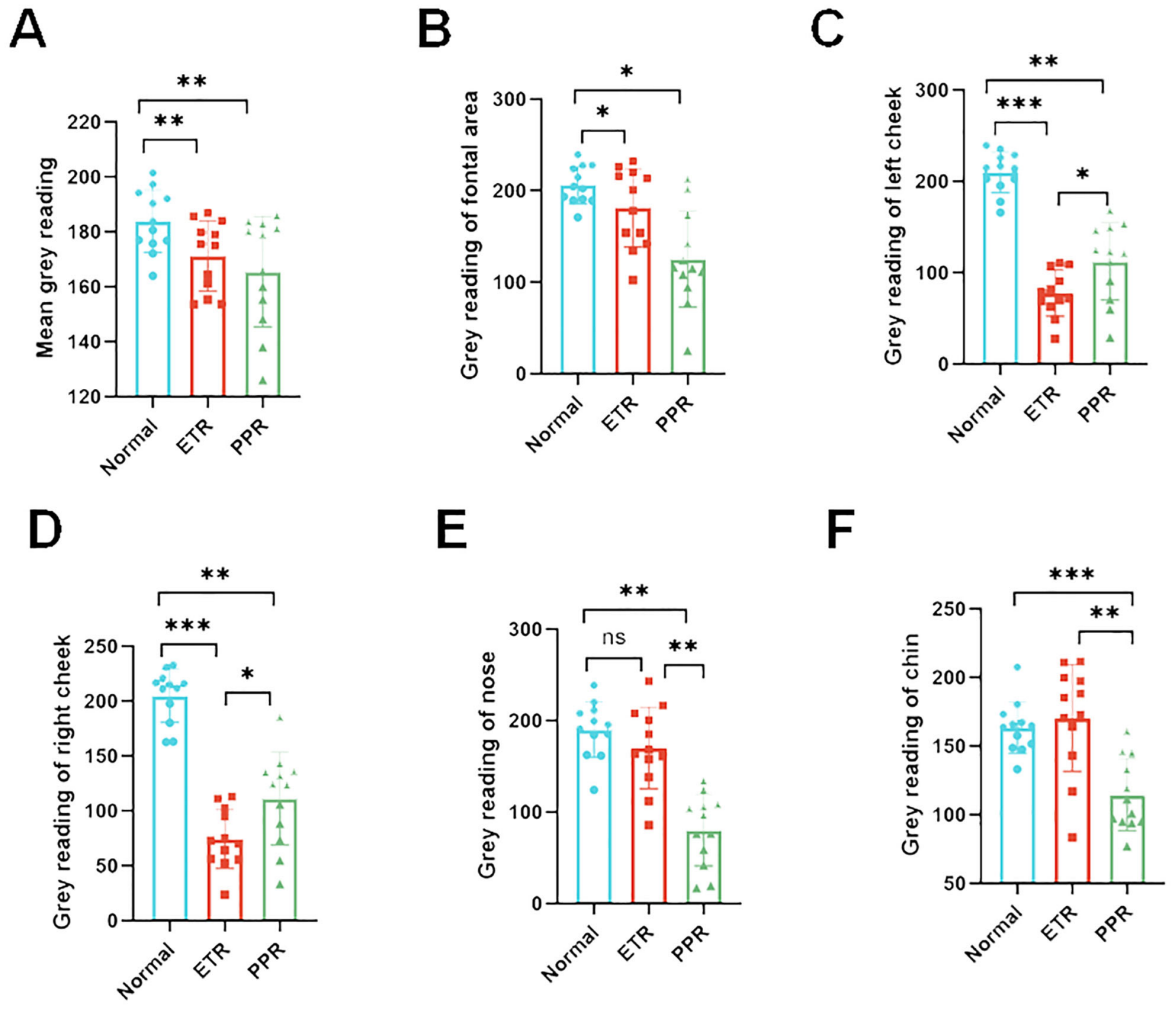
Gray value analysis across various facial regions depicting inflammation intensity. The inflammation distribution is consistent across all groups with some variations. **(A)** Overall facial gray values (vs. control, ETR, and PPR *p* < 0.01), **(B)** forehead (vs. control, ETR, and PPR *p* < 0.05), **(C)** left cheek (vs. control and ETR *p* < 0.001, PPR *p* < 0.01, between ETR and PPR, *p* < 0.05), **(D)** right cheek (vs. control and ETR *p* < 0.001, PPR *p* < 0.01, between ETR and PPR, *p* < 0.05), **(E)** nose (vs. control, not significant in ETR and PPR *p* < 0.01, between ETR and PPR *p* < 0.01), **(F)** and chin (vs. control, not significant in ETR *p* > 0.05, PPR *p* < 0.001, between ETR and PPR, *p* < 0.01). *P<0.05, **P<0.01, ***P<0.001.

In the case of the left and right cheeks, a significant decrease in gray values was observed in the ETR group compared to the normal group (*p* < 0.001), with the PPR group also showing a decrease (*p* < 0.01). Notably, the decrease in the ETR group was more pronounced than that in the PPR group (*p* < 0.05). On the nose, the ETR group did not show a significant change in gray values, and the PPR group demonstrated a notable decrease in gray values compared to both the normal and ETR groups (*p* < 0.01). On the chin, the ETR group did not show a significant change in gray values and the PPR group demonstrated a significant reduction in gray values, showing a marked difference from the normal (*p* < 0.001) and ETR group (*p* < 0.01, **Figure 6**).

### Detection of plasma biomarkers in different groups

**Figure 7:**
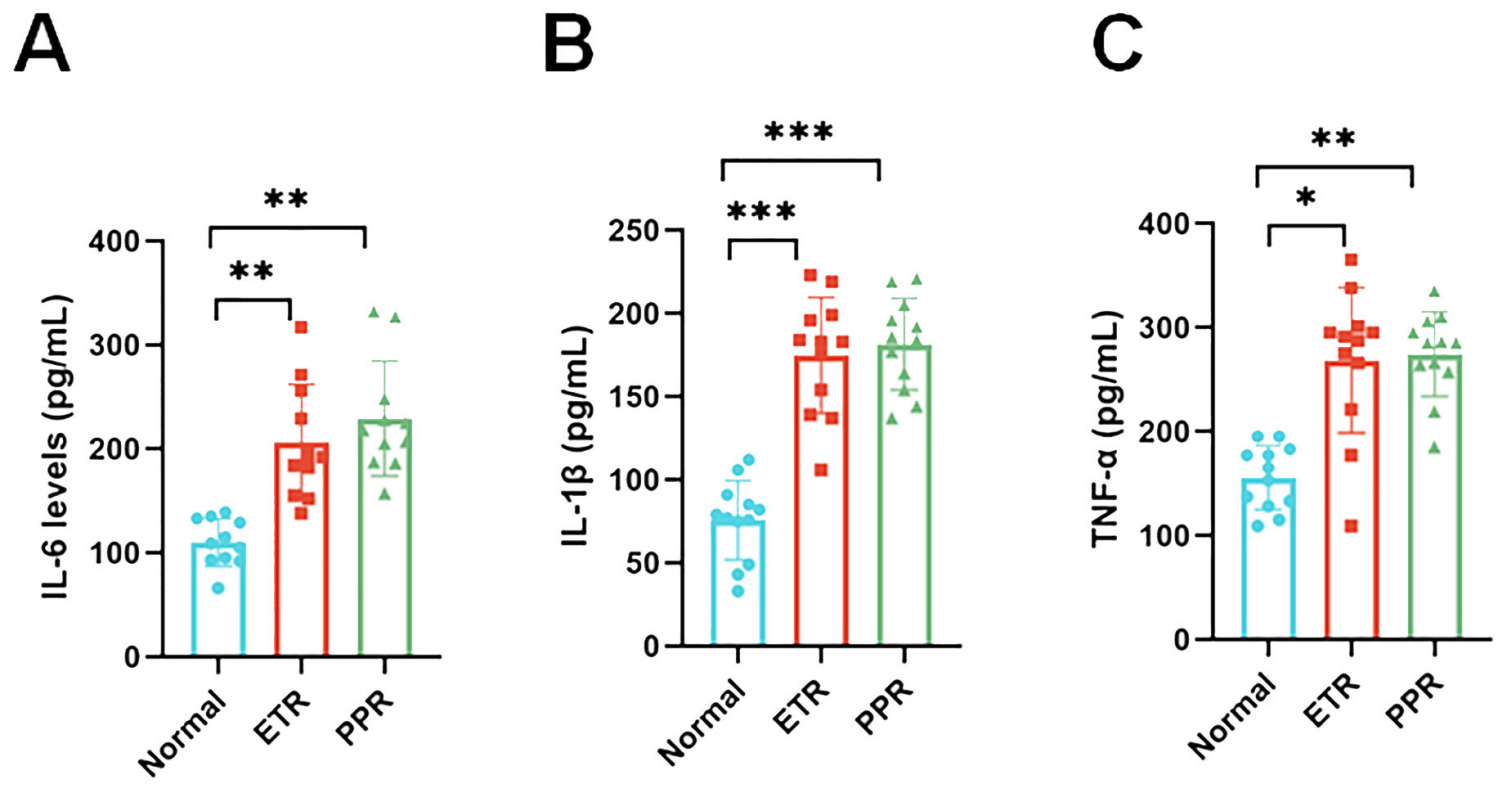
Serum pro-inflammatory cytokine concentration levels in the control, ETR, and PPR groups suggest systemic inflammation [interleukin-6 (IL-6): *p* < 0.01 ETR vs. control, *p* < 0.01 PPR vs. control; interleukin-1 beta (IL-1β): *p* < 0.001 for both ETR and PPR vs. control]. Tumor necrosis factor-alpha (TNF-α): *p* < 0.05 ETR vs. control, *p* < 0.01 PPR vs. control. *P<0.05, **P<0.01, ***P<0.001.

Plasma biomarkers have been instrumental in validating the inflammatory profiles within different groups. Among these, the IL-6 levels in both the ETR and PPR groups were significantly elevated compared to the normal group, showcasing a marked statistical difference (*p* < 0.01). This suggests a systemic inflammatory response that correlates with the clinical presentations of these conditions. Similarly, IL-1β levels were notably higher in both the ETR and PPR groups when compared to the normal group, with the difference reaching a high level of statistical significance (*p* < 0.001). This further supports the notion of an enhanced inflammatory cascade in these rosacea subtypes. Moreover, TNF-α, another critical inflammatory mediator, was found to be increased in both the ETR and PPR groups relative to the normal group. These increases for the ETR group (*p* < 0.05) and the PPR group (*p* < 0.01) are statistically significant (**Figure 7**).

## Discussion

Rosacea has a high incidence, a high impact on quality of life, and insufficient existing diagnostic techniques. Our results indicate that LSCI can be used for the non-invasive adjunct diagnosis of rosacea. In this study, both ETR and PPR groups showed increased average blood perfusion and facial inflammation intensity compared to controls, with statistically significant differences. Plasma biomarkers IL-6, IL-1β, and TNF-α were significantly elevated in both ETR and PPR groups compared to controls. This confirms that, compared to healthy individuals, patients with rosacea indeed exhibit characteristics such as facial vasodilation, increased blood flow, and concurrent inflammatory responses.

Think View employs multispectral technology, such as polarized and NIR imaging, to capture high-resolution skin images, highlighting features like blood cell behavior invisible to the naked eye (23). This device, used for detecting vascular skin diseases, benefits from NIR spectroscopy, which assesses blood hemoglobin levels, useful for examining tissue perfusion (24). Near-infrared Raman spectroscopy (NIRS) penetrates deep tissue, allowing it to measure regional blood oxygen saturation (rSO_2_) in the microcirculation, thereby providing a better indication of local tissue perfusion (25). The data are largely based on computers and technicians, which may introduce some bias in data presentation. Despite this, it is still widely applied in clinical settings. In the current study, we analyzed the different areas of rosacea on patients’ faces using gray values. Compared to the normal group, the gray values of the ETR group showed a marked decrease, and the PPR group demonstrated a similar decrease. These differences are statistically significant (*p* < 0.05) when compared to the normal group. There is no statistically significant difference between the ETR and PPR groups. We also found that the more severe the facial lesions are, the lower are the gray values. In comparisons of different areas within each group, the results reflected the objective degree of skin damage and highlighted the differences in affected areas between different types of rosacea. ETR more frequently involved the cheeks, whereas PPR was more commonly found in the traditional T-zone (forehead, nose, and chin). This study supplements our understanding of the objective damage caused by skin lesions and the variations in affected areas among different rosacea subtypes.

LSCI is a cutting-edge technology in our study, capable of adjunct diagnosis if more population-based studies are performed, and it has the potential to monitor blood flow in rosacea. Previous studies have shown that LSCI can be used in various clinical scenarios, including wound and cerebral perfusion monitoring (26–28). Kanemura et al. confirmed the assessment of skin inflammation using NIRS with a 785-nm laser, combined with artificial intelligence (AI) analysis in an animal model (29). However, no study to date has correlated LSCI with blood flow in rosacea. Our research indicates that LSCI can dynamically monitor different areas of rosacea simultaneously. To explore differences in affected areas among patients with rosacea, we performed a thorough investigation, focusing on the comparison of specific areas. We divided the face into the forehead, cheeks, nose, and chin. These areas reflect differences in facial sebum secretion, with the forehead, nose, and chin being high sebum secretion zones, while the cheeks are relatively low sebum secretion areas. Our experimental data, as shown in **Figure 5**, indicated that ETR affects the cheeks, forehead, nose, and chin, with the highest increase in blood flow observed in the cheek area. PPR lesions were more pronounced in the forehead, nose, and chin (traditionally known as the T-zone, areas with increased sebum secretion). In this study, although the forehead, nose, and chin were not statistically significant compared with ETR, they correlated with individual differences and small samples were considered. If the most severe patients in the original data were excluded, there were differences between the groups. However, because the other parts of the patient were not special, they were not included, resulting in no difference between the groups, although the cheek area could also be affected but not as significantly as in the ETR group. This suggests different sources of inflammation, as previous studies have indicated that prominent involvement of the cheek area might be more related to neurovascular stimulation by triggering factors, while involvement of the facial T-zone might be associated with increased sebum secretion and microbial infections such as *Demodex* mites. This study pioneers the use of LSCI for full facial and regional blood flow assessment in rosacea, offering new, non-invasive diagnostic possibilities. However, there are still some limitations to the current study. LSCI provides semi-quantitative data, as its units are not true measures of blood flow—measuring velocity or flux instead. There is no significant difference in systolic BP (*p* > 0.05 for controls and ETR, *p* > 0.05 for controls and PPR) and diastolic BP (*p* > 0.05 for controls and ETR, *p* > 0.05 for controls and PPR). It is of critical importance to interpret the LSCI data with arterial blood pressure (ABP) because the skin is less regulated by factors such as autoregulation. A change in ABP can change blood flow without associated vasodilation. However, in the current study, we did not assess ABP for the following reasons. Firstly, all participants were asked to avoid vigorous exercise, emotional fluctuations, smoking, drinking coffee, and other factors that might aggravate erythema 30 min before testing and to rest quietly for 10 min before Think View and LSCI imaging. Therefore, there might be no blood pressure fluctuation before diagnosis. Secondly, the diagnosis duration of Think View is approximately 2–3 s (not including the photographic delay period), and the diagnosis duration for LSCI is approximately 4–5 s. During such a short time, LSCI could hardly induce the fluctuation of ABP. Thirdly, and more importantly, LSCI does not heat the skin and it is just a detection facility without directly contacting the skin; therefore, it will not increase the blood flow of participants. Lastly, as all the participants received LSCI diagnosis, not therapy, there will be no change in ABP for all of them. In this research, according to the severity of the diseases, we included paired patients (four patients with severe rosacea, four patients with moderate rosacea, and four patients with mild rosacea). We further analyzed the underlying flow over telangiectasia by comparing the mean perfusion volume of the same areas among the patients with mild, moderate, and severe rosacea. For the patients with ETR, no matter whether they belong to the mild, moderate, or severe category, there are significant differences in the overall face, left cheek, right cheek, nose, and chin areas. However, for the patients with PPR, only the nose areas and chin areas have significant differences among different categories of patients (mild, moderate, or severe). Therefore, the disease progression could be identified by the severity. It is unfortunate that the patients did not go to the clinics following therapy; thus, the disease trajectory is missing. Meanwhile, the microvascular dysfunction was not assessed by ultrasound in this study due to the lack of suitable ultrasound facility for dermatology. It is one limitation of this study.

Rosacea is a chronic inflammatory disease involving deregulation of the immune system. The link between LL-37 and rosacea was demonstrated by Yamasaki et al. (30) in an inflammatory skin mouse model induced by the injection of LL-37, which developed telangiectasia, erythema, and inflammation. Using the same model, Yuan et al. (31) showed that the skin expressed higher levels of IL-6, IL-1β, and TNF-α compared to the control group. Activation of immune-mediated inflammatory pathways appears central to the pathogenesis of rosacea and involves the coordinated activity of several cell types, such as mast cells and macrophages, and the release of proinflammatory mediators, such as IL-6, IL-1β, and TNF-α (32, 33). Inhibition of these inflammatory pathways is correlated with clinical improvement. IL-1β, IL-6, and TNF-α are common inflammatory factors that reflect the body’s inflammation levels, changing in response to infections, sepsis, or other inflammatory triggers (34, 35). Therefore, cytokines in serum represent the whole body and are not specific to facial locations. In addition, as cytokines have kinetics, the serums were collected on the day of Think View and LSCI diagnosis, to make sure that there is less fluctuation of cytokines for each patient. We excluded patients with acute inflammation, infection, tumors, and other diseases to ensure accurate detection of these three inflammatory factors, confirming the inflammatory changes in patients with rosacea. Additionally, while measuring inflammatory factors in facial skin tissue might be more accurate, it requires facial surgery to obtain samples, which is challenging due to the potential for facial disfigurement.

Overall, our results demonstrate that LSCI is a novel and potentially groundbreaking technology for the adjunct diagnosis of rosacea. This cutting-edge technology can reflect the differences in blood perfusion across various facial areas, providing a data foundation, and has the potential to ultimately assess severity, leading to the early diagnosis and treatment of rosacea at its initial stages, even before its onset. The integration of LSCI technology into the study of rosacea represents a significant advancement in the non-invasive evaluation and understanding of this complex dermatological condition (24, 36). By offering a detailed and dynamic visual representation of blood flow and perfusion across affected facial areas, LSCI transcends the limitations of previous diagnostic methods (37, 38). Its ability to provide semi-quantitative data for blood velocity or flux, facial erythema, and vascular abnormalities facilitates a deeper insight into the pathophysiology of rosacea. Additionally, the capability of LSCI to capture subtle changes in microvascular blood flow equips researchers and clinicians with a potential tool for the early detection of rosacea, potentially before the onset of visible clinical symptoms.

LSCI can improve the accuracy of displaying the area and extent of facial erythema. This study found that the results between LSCI and the computer imaging system were closely related. When measuring facial erythema, the deeper the erythema color, the higher the LSCI value, and the lower the grayscale value of the multispectral imaging system’s NIR images. This correlation confirms the severity of rosacea using these two technologies. This may help design a more objective and quantitative system to evaluate the severity of rosacea. However, a large amount of data are still needed in the future to provide references for stratifying severity.

Additionally, the non-contact nature of LSCI is a critical advantage in studying rosacea, a condition often characterized by heightened skin sensitivity. Techniques requiring direct skin contact or invasive procedures can aggravate the skin, potentially skewing diagnostic results or worsening the patient’s condition. LSCI circumvents these issues, offering a safe, comfortable, and accurate means of assessing facial blood flow dynamics without direct physical intervention. This aspect is particularly beneficial for longitudinal studies.

This innovative tool can visualize and measure facial blood flow dynamics in real time, which is helpful for investigating the underlying mechanisms of the disease. By enabling detailed analysis of how various factors—such as environmental triggers, psychological stress, and microbial colonization—impact facial blood flow and erythema, LSCI can help identify new pathophysiological pathways and targets for therapeutic intervention for providing a data foundation for the diagnosis and treatment of rosacea.

Based on the test results, personalized treatment can be considered for patients. Patients with significantly increased blood perfusion in the cheeks, as indicated by LSCI, and significantly reduced grayscale values in the cheeks, who clinically present with flushing, can be treated with therapies targeting flushing. These treatments may include anti-inflammatory and immunosuppressive therapy with hydroxychloroquine and β-adrenergic receptor inhibition with carvedilol (39, 40). Patients with significantly elevated LSCI and significantly reduced grayscale values in the nose, forehead, and chin, mainly due to the significant contribution of sebaceous glands in these areas, the clinical manifestations being papules and pustules, can try to use antimicrobial and anti-inflammatory treatment regimens, such as doxycycline-based drug treatments (41).

Additionally, ETR and PPR affected multiple facial areas with overlaps, further indicating the overlap and transition between different subtypes of rosacea. These findings not only underscore the heterogeneity of rosacea but also show the simultaneous occurrence of more than one subtype and the potential progression from one subtype to another. This aligns with the latest classification approach for rosacea, which is based on symptomatic phenotypes rather than simple subtype distinctions.

The study’s small sample size is acknowledged as a limitation, yet it is posited that the emerging technology’s novelty grants a high degree of innovation to the findings. Even with fewer participants, consistent trends and coherent conclusions were observed, indicating reliable data that may predict larger population behaviors. The difficulty in conducting invasive immunological testing across various facial regions further justifies the methodological approach.

LSCI in the detection application of rosacea is an exploratory study, demonstrating its feasibility in measuring blood flow in patients with rosacea. The data to some extent proved the differences and overlaps in the affected sites and subtypes of patients with rosacea. We will expand the sample size and establish a multi-center study for LSCI research data, combined with AI. With the combination of AI and more data, LSCI will be a novel technology in the future and explore the changes in patients with rosacea under different influencing factors, facilitating a deeper insight into the pathophysiology of rosacea, to identify disease progression and map disease trajectory, and directly assess vascular dysfunction and precision outcomes, aiming for personalized diagnosis and treatment.

## Data Availability

The raw data supporting the conclusions of this article will be made available by the authors, without undue reservation.
