# Supplementary figures and images for: Exploring the efficacy of laser speckle contrast imaging in the stratified diagnosis of rosacea: a quantitative analysis of facial blood flow dynamics across varied regions
Source: Front Immunol. 2024 Aug 23;15:1419005. doi: 10.3389/fimmu.2024.1419005 (PMC11377348; doi:10.3389/fimmu.2024.1419005)

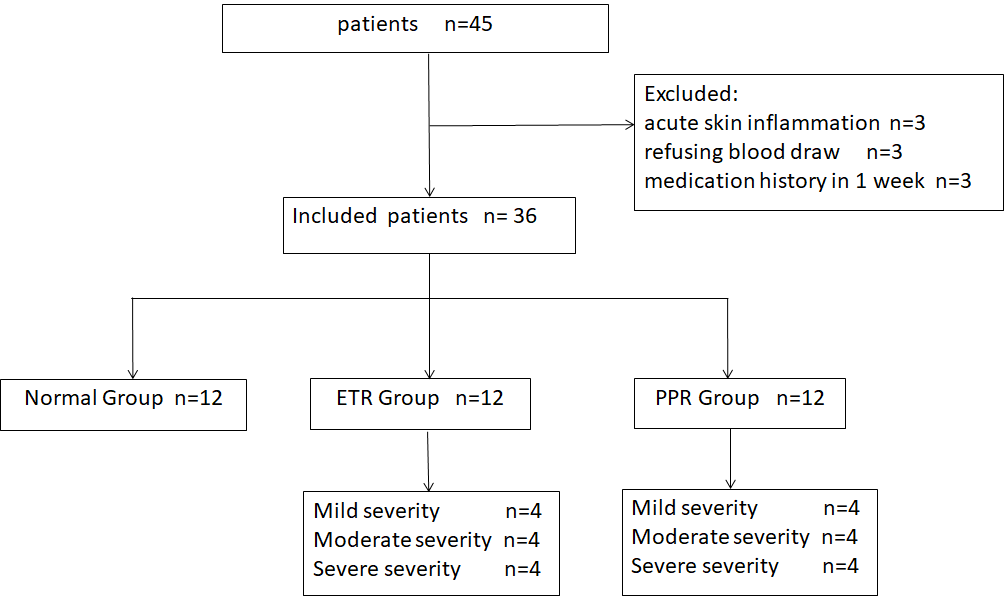

Supplement: Supplementary file 1 [file Image1.tif]
